# The evaluation of podiatrists, with knowledge and training in diagnostic musculoskeletal ultrasound, to describe sonographic images of diabetic foot wounds in the United Kingdom and Australia

**DOI:** 10.1186/s13047-022-00511-0

**Published:** 2022-01-25

**Authors:** Charlotte Dando, Georgia Lane, Catherine Bowen, Frances Henshaw

**Affiliations:** 1 School of Health Sciences, Faculty of Environmental and Life Sciences, University of Southampton, Southampton, UK; 2 Podiatry Department, Solent NHS Trust, Southampton, UK; 3 Centre for Sport, Exercise and Osteoarthritis Versus Arthritis, University of Southampton, Southampton, UK; 4 School of Medicine, Western Sydney University, Penrith, Australia

**Keywords:** Diabetes mellitus, Diabetic foot ulcer, Ultrasound, Podiatrist, Sonography

## Abstract

**Aims:**

Currently, wound management decisions are based largely on visual observations such as photographs, descriptors or measurements which can lack detail and do not always capture the sub-wound area. A previous case series suggests that there is benefit in using ultrasound imaging (USI) to evaluate diabetic foot ulcers (DFU) at point-of-care, however no guidance exists to inform its use. This scoping exercise explores the capacity of podiatrists with experience in interpreting musculoskeletal structures using USI to interpret sonographic images of DFU.

**Methods:**

Following a short briefing session, podiatrists with previous musculoskeletal (MSK) USI training were asked to review and report on previously recorded static sonographic images (*n* = 8) of active DFU. Content analysis was utilised to identify recurring keywords within the podiatrists’ reports which were coded and assigned to categories to gain context to the data.

**Results:**

Seven podiatrists participated in the study. Four categories were constructed for the purposes of analysis:

1. Frequency of reporting, 2) Language used in reporting, 3) Observations, 4) Clinical impression

Frequently, the reported findings between podiatrists were found to be similar, especially those related to bone morphology. However greater variability was seen in the reporting of wound specific soft-tissue observations.

**Conclusion:**

This scoping exercise has shown that podiatrists can translate their existing USI skills to make rudimentary reports on clinical findings in DFU. All participants were consistently able to identify and describe characteristics associated with DFU from a single b mode static wound ultrasound image. Findings from this investigation can be used as a foundation for further work to establish accuracy and reliability to validate DFU sonography. In conjunction the development of protocols and training materials will enable the adoption of USI to assess DFU in clinical practice. This will in turn, contribute to improved patient care and establish a new paradigm for wound surveillance which is translatable to other wound types.

## Background

Diabetes related foot ulcers (DFU) are one of the most common and serious complications, occurring in up to 30% of people with diabetes [1] and frequently DFU result in amputation. It is estimated that 10–25% of people living with diabetes, at any one time will develop a DFU [2]. DFU are associated with a significant burden in terms of morbidity, mortality as well as socio-economic costs [3]. Recurrence rates of healed ulcers are high, up to 65% within five years, thus further evidencing the devastating consequences of this complication [4].

DFU are characterised by their chronicity, hyper granulation, persistent inflammation and reduced healing capacity [5]. Poor outcomes including delayed healing and amputation are often related to neuropathy, the presence of peripheral arterial disease as well as wound depth and infection severity [6–12].

Wound assessment can be defined as: ‘using observation, questioning, and clinical investigations to enable selection of appropriate therapeutic strategies in order to achieve clinical goals such as healing and improved quality of life’ [13]. A number of techniques exist to assess wound morphology, such as photography, digital planimetry, acetate tracing and more recently laser or structured light approaches. However, these provide limited information as they do not depict the sub-wound area and therefore the full extent of the wound is not captured. The probe-to-bone test is commonly used to determine wound depth as well as having utility as a clinical test for osteomyelitis, but its accuracy has been questioned in clinical settings where the prevalence of osteomyelitis is low [14].

Imaging modalities exist which can be applied to interrogate wounds in three dimensions (3D) such as VeVMD (Vista Medical, Winnipeg, Manitoba, Canada) for wound volume; Tissue Analytics, (Baltimore, Maryland) for tissue composition. However, these measurement techniques still do not capture all facets of the sub wound area such as bone morphology, tissue stiffness and vascularity which might provide a more complete picture of the wound status.

X-ray is helpful to preclude osteomyelitis or bone fragments that might be hindering the healing process and is readily available, inexpensive and provides quality resolution of bones [15]. Yet, in cases of early OM, the rate and accuracy of detection by X-ray is as low as 50%, largely because soft tissues are not adequately captured [16].

Good evidence exists to support the diagnostic accuracy of magnetic resonance imaging (MRI), computerised tomography (CT) and positron emission tomography (PET) in evaluating DFU [17, 18]. Diagnostic imaging is often used to inform surgical planning, infection and Charcot Neuroarthropathy management in relation to diabetic foot complications [19, 20]. Whilst these imaging systems are able to accurately map an entire wound and indicate the presence of pathology they are not readily available, require specialist referral and are expensive [21]. Hence, there is currently no system employed in routine practice that can capture the full extent of a wound at point of care.

Currently, diagnostic MSK ultrasound imaging (USI) is used to assess soft tissue structures of the foot for signs of inflammation, tears and ruptures [22]. It also has the capability to assess vascularity (power Doppler) and tissue stiffness (elastography). This information, in combination with a medical history, can be used to help support clinical decision-making plus and as an educational tool to more comprehensively inform a person of their condition. Notably, of podiatrists completing a recent international survey (*n* = 239), USI is expanding in Europe (Ireland (0.43%; Italy 0.86%; Malta 1.29%; Spain 31.47%; Netherlands 11.64%; UK 37.07%) and also Canada (12.5%), Australia (3.5%), South Africa (0.45%), USA (0.43%) and Kenya (0.43%) [23]. Contrary to this there is no legal requirement for podiatrists to hold a recognised ultrasound qualification in order to practice as a sonographer in the UK or Australia and sonography is not recognised as a profession by the Health and Care Professions Council (HCPC) nor the Australian Health Practitioner Regulation Agency (AHPRA). Variability in training pathways for USI exists such that the level of USI competency those podiatrists have is not known [23]. However it is known that the majority of work in this field by podiatrists has been conducted in people with MSK injury or people with inflammatory conditions [22]. USI has not been extensively tested in populations with DFU and wound USI is not part of current USI learning curriculums in either Australia or the UK. Apart from a small case series, no guidance exists for USI use to assess wounds exists [24].

Given that current tools for wound assessment are unable to comprehensively map the sub-wound space at point-of-care, in the concept of utilising USI in clinic to support empirical thinking when evaluating DFU has appeal. USI has been shown to be portable, safe and non-invasive to the person receiving the scan, as well having low costs to running and maintaining the equipment [22, 23]. Thus, this technology could be deployed at point of care to review characteristics of tissue structure including depth, tracking or sinuses, monitoring vascularity, stiffness and to identify bone and tendon changes. Review of tissue characteristics would enable a more precise management plan and support monitoring of the wound healing status.

To date, evidence to support the use of USI for wound assessment is scarce. By understanding the characteristics of DFU as seen under USI and appraising the ability of podiatrists with MSK USI training to transfer their skills to determine whether the use of USI in DFU assessment is feasible. This scoping exercise explores the idea that podiatrists who have experience and knowledge in interpreting MSK structures under ultrasound may also be able to interpret sonographic images of DFU. If podiatrists are able to interpret wound ultrasounds, there is potential to further research in this topic area and ultimately improve the way that wounds are assessed at point-of-care.

## Methods

A scoping exercise was designed to capture the descriptors used to identify tissues in DFU and podiatrists’ comments drawn from reviewing a static sonographic image of active DFU.

The aims of the scoping exercise were:

1. To capture the language, phrases or descriptors used by podiatrists to review DFU under b mode static images.
2. To explore whether podiatrists were able to clinically comment on DFU under b mode static images
3. To identify which specific themes were used to identify DFU characteristics from b mode static images.

### Participants

Participants were a convenience sample of podiatrists derived from all podiatrists at the two facilities where the ethical approval for the study had been granted in Australia (Sydney Metropolitan region) and the United Kingdom (Wessex region). This equated to 100% of known podiatrists in each setting who had the requisite training.

Australia (Sydney Metropolitan region) and the United Kingdom (Wessex region), with different levels of experience and knowledge in identifying DFU and foot/ankle anatomy from sonographic images (Table 1). No podiatrists declined to take part. Approval to conduct this evaluation was given by Solent NHS Trust, Research, and Improvement Team and **‘**HREC/17/HAWKE/61’ in Australia. Participants were asked to provide written informed consent and proof of ultrasound training.

- either had gained the MSK Consortium for the Accreditation of Sonographic Education (CASE) accreditation
- or had attended a training short course in diagnostic MSK ultrasound.

**Table 1:** Training levels and experience of podiatrists using USI

Participants Code	Level of Training (formal/informal)	Experience levels of ultrasound (in years)	Number of patients scanned a month (approximately)	Purpose USI skill used for
AUS	2 day course	1	0	clinical and research
AUS	2 day course	3	0	clinical and research
AUS	2 day course	3	0	clinical and research
AUS	2 day course	3	4 scans/ month	clinical and research
UKJ1	CASE Accredited	4	20 scans/ month	clinical
UKM2	Short course	1	0	research
UKC3	CASE Accredited	3	3 scans/month	clinical and research

### Training materials

**Fig. 1:**
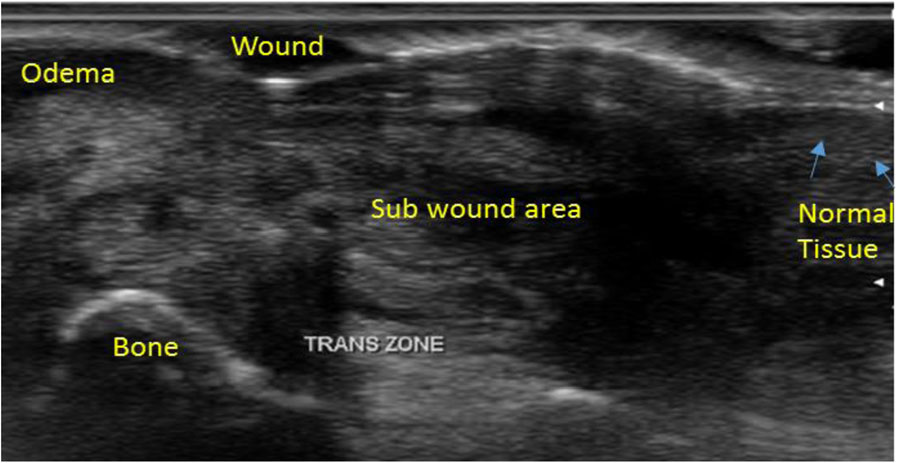
Annotated image to orientate participants to the features of a wound as seen by USI in the transverse plane

Participants used email, Microsoft Word and Power Point programmes in order to open and access the E-documents in addition to the submission of their reports. An online introductory lecture, outlining the features of wounds as seen under ultrasound was provided to all participants as an orientation activity. This was followed by a short tutorial where participants were able to practice and develop their skills by studying four different ultrasound wound images. Anatomy of the skin and subcutaneous tissues were described and demonstrated with figures (Fig. 1). This approach ensured that a baseline understanding was achieved before the review process. Each participant had received the same access to information and had a rudimentary understanding of wound characteristics as seen on ultrasound. Training materials were developed through the consensus of two podiatrists (FH and CD), FH has imaged more than 100 wounds and CD has extensive experience in MSK USI.

### Data collection process

Upon completion of the orientation activity, participants gave informed consent to partake in the scoping exercise, which was approved by Solent NHS Trust, Research and Improvement Team and **‘**HREC/17/HAWKE/61’ in Australia. Sonographic images used in the exercise were anonymised and written consent had been obtained, prior to the scoping exercise, from the patients to use these images in further research/project activities and for publication.

### Intervention

Once consent was obtained, participants received an email with the scoping exercise instructions and data collection forms. A reminder email was sent to the participants one week after the initial email was sent. Each participant was asked to write a report on eight ultrasound images of DFU, they were given autonomy to comment on what they felt was important to note. The location of the DFU in the image was described and marked as a reference point.

All data was anonymised and remained at group level. Data from the reports associated with each image was coded into themes and analysed by the study team. Content analysis was utilised to identify recurring keywords within the reports which were coded and assigned to categories to gain context to the data. Analysis of ultrasound reports enabled a direct examination of the content of each participants written report, thus avoiding biases of recall. Sample selection from the written reports of each participant followed a multistage process [25] with the report as the unit of analysis. To ensure basic stability of the coding scheme, each report was analysed by two coders independently (FH and GL). The coders were trained in content analysis over a 3 week period using reports that were not included in this sample to test reproducibility. Disagreements were resolved by consensus and to ensure validity all disagreements were documented prior to achieving a consensus. The resulting data was coded and assigned to categories to gain context to the data.

## Results

Seven podiatrists consented to participate. All had experience in using USI that ranged from 1 years to 4 years (Table 1).

Four categories were constructed:

1. Frequency of reporting, 2) Language used in reporting, 3) observations, 4) Clinical impression

### Frequency of reporting

Wounds were broadly defined into 3 categories based on their location: non-weight bearing toe (1); plantar forefoot (4); and midfoot (3). All seven participants prepared an individual report for each of the eight wounds. The reports were analysed by two investigators and coded. Four distinct themes were derived, identifying key structures within the wound location. Firstly, the wound and sub wound area (break in continuity of skin/tissue) and this was most commonly reported upon (52 cases). Secondly, the underlying bone (50 cases). Thirdly, the peri wound area (the area of tissue surrounding a wound) (19 cases) and fourthly, the tendon structure and composition relative to other soft tissue structures was less frequently reported (13 cases). One participant did not provide commentary on tendons in any of their reports whilst others did not describe the peri-wound area within their reports (Table 2).

**Table 2:** Repetition of reported structures by participants

	0 reports	1report	2 reports	3 reports	4 reports	5 reports	6 reports	7 reports	Total Reports
Periwound	3		2			3			**19**
Wound						1	2	5	**52**
Tendon	4	4	1	1	1				**13**
Bone						2	3	4	**50**

In terms of frequency, the forefoot wounds were most comprehensively reported upon with an average of 18 reports across the four domains made per wound, followed by the midfoot, with an average of 15.67 reports and the single dorsal forefoot (toe ulcer) having 14 reports (Table 3).

**Table 3:** Frequency of reporting

	Plantar FF				Midfoot			Dorsal FF
Wound #	1	4	6	8	2	5	7	3
Periwound	5	5	2	0	0	0	5	2
Wound	5	7	7	7	6	7	7	6
Tendon	3	1	4	2	0	1	1	1
Bone	6	5	6	7	6	7	7	5
**Total Reports**	**19**	**18**	**19**	**16**	**12**	**15**	**20**	**14**
**Average**				**18**			**15.67**	**14**

All participants reported their observational findings using a superficial to deep approach to documenting anatomical structures. This started with skin, wound, tendon, soft tissue and then bone before, in some cases, stating their clinical impression.

### Language used in reporting

The language used for reporting was examined and coded into four themes: soft tissue, morphology, bone and specific sonography terms. 15 phrases were identified which described the soft tissues; 17 phrases described bone pathology and structure. 25 phrases pertained to morphology, defined as ‘a particular form, shape or structure’. Whilst 14 sonographic specific terms were identified. The most commonly reported terms in each of these themes is shown in Table 4.

**Table 4:** Themes and commonly reported terms

Theme	Most commonly used terms and frequency
Soft tissue	Wound/ulceration (151); soft tissue (45), tendon (28), oedema (21), skin (13)
Morphology	Irregular (17), sinus (16), tracking (10), regular (9), Shallow (6)
Bone	Bone (84), joint (23), metatarsal head (19), osteomyelitis (19), styloid process (7)
Sonography terms	Deep/depth (48), hypoechoic (45), transverse (16), diameter (15), measuring (13)

### Observations

For wound and sub wound soft tissue areas identified on the US images, six participants indicated a sinus in wound image one and also identified that a hypoechoic area could be seen adjacent to wound images two, five, seven and eight. However, aside from these observations, a high degree of variability between participants was also recognised in the reporting within this theme or the wound and sub wound space. For example in wound image four has phrases documenting the presence of bone, tendon or soft tissue that was reported as ‘normal’; ‘hyperechoic’; ‘artefact’ or ‘foreign body’. Whilst the sub wound area of wound image three was reported as hypoechoic by four participants and as heterogeneous by a further two.

The periwound area was described in five of the eight images with between two and five participants making reference to it. In each of the five cases the presence of periwound oedema was noted– referred to variously as ‘anechoic fluid in the shape of wings’; ‘hypoechoic sub-dermal oedema’; ‘periwound oedema’.

Bone appearance was another theme where distinct terms were used to report visual findings. Bone structures were visualised on 7 out of the 8 ultrasound b mode images used for the exercise. The majority (six out of seven) of participants described the bone appearance in similar terms, deeming it ‘regular’ in three cases and ‘irregular’ in four cases. Participants’ reporting was less definitive for one image, where five participants reported ‘regular’ bone echotexture; one was ‘unsure of bone margin’ and the final reported that ‘it is likely that there is osseous involvement. A high suspicion of osteomyelitis should remain’. The mid foot wound images (*n* = 3) all had abnormal bony architecture. Two DFU were as a result of osseous changes associated with a previous midfoot Charcot’s Neuroarthropathy and another had undergone a surgical debridement of the shaft of the 5th metatarsal. Whilst the participants consistently reported the bony irregularity of the Charcot joints (12 out of 13 reports), they did not correctly specify the cause (Charcot’s) in the majority of cases (12 out of 13). Other diagnoses including gout, osteomyelitis and osteophytosis were documented. Whilst seven out of seven participants reported the debrided 5th metatarsal as ‘regular’ and one noted that the bone was unusually hyperechoic, no participants were able to identify the surgical debridement.

Tendon was the least commonly reported of the four themes, with only 13 comments about tendon status made out of a possible 56 reports, and a maximum of four comments per image. There was consensus amongst three participants, who reported on the tendon in wound image one, that the wound extended to/involved the tendon. However, four participants described the tendon in wound image six, variously reporting the flexor hallucis longus (FHL) tendon as: ‘looks attached, homogenous with a uniformed fibrillar structure’; ‘distal attachment of the FHL tendon showing some thickening and likely calcific enthesopathy’; ‘no involvement of underlying tendon’; ‘partially hypoechoic tendon structure’.

### Clinical impression

In 11 out of the 56 reports that were made, no clinical impression was given. The most frequently reported clinical impression pertained to the presence of OM (18 reports). Other impressions included suspicion of a ganglionic cyst (1); abscess (1); uric acid crystallisation (1); foreign body (2); joint dislocation/degeneration (2); enthesitis (1); and tendon rupture (1) illustrating that a high degree of variability existed between participants when providing their clinical impression.

Several patterns of reporting DFU using ultrasound images were noted: Participants wrote their reports using the ‘superficial to deep’ approach. Frequently, comments referred to the skin and open wound, followed by the underlying soft tissue and bone. Most participants, 45 out of 56, provided a clinical impression or action pertaining to the clinical question posed. Three participants commented that it was challenging to report static images.

## Discussion

The purpose of this current scoping exercise was to determine the extent to which podiatrists with MSK ultrasound training were able to translate their knowledge to report on the characteristics of DFU. Podiatrists with training in using MSK USI were able to identify and describe DFU characteristics from a single b mode static wound image and broadly there was consistency in reported findings for the images. Where more unusual features were apparent such as heterogeneity in soft tissues, the podiatrists were less consistent in providing clinical impressions. Standardised guidelines for clinical report writing exist for USI and are a fundamental part of MSK USI clinical practice [26, 27]. It would appear, in this sample, that USI guidelines were used as a basis for the purposes of wound reporting. This would suggest that some aspects of the skill surrounding professional working standards, governance and safety will be similar, if not the same for DRU and MSK US examination.

Through this scoping exercise, potential barriers to using USI for wound assessment have been presented in addition to future opportunities that could be implemented to improve current thinking and process.

### Barriers to accurate reporting

The US report summarises crucial findings in plain language and plays a central role in communicating these findings to colleagues [26]. However, the process of generating impressions is challenging when no reference data exists to inform decisions. To our knowledge, no sonographic protocols exist for imaging DFU which means that it is not possible to verify the accuracy of reports provided by the participants.

Three participants indicated that they might be able to make a more definitive conclusion if able to interrogate the wound with real time, dynamic imaging, rather than relying on a single static image. Improper scanning techniques can introduce artefacts into static images, which if not appreciated by the reporting clinician may lead to incorrect interpretation [28]. In real-time US, image formation is automatic, and new images are continuously produced many times per second. Freely moving the transducer in real time can more easily exclude anomalies such as artefact leading to more accurate reporting [29]. This is something that should be considered in future research to ensure suitable applicability to clinical practice.

Currently, there is minimal evidence to support the use of ultrasound to enhance clinical decision making in wound management. The development of clinical guidance documents like those already available for musculoskeletal care, would enable podiatrists to incorporate wound sonography into their practice.

### Strategies to improve reporting accuracy

The results of this scoping exercise show there is potential for US to be used to support clinical decision making in populations with DFU. From the content analysis of the wound ultrasound reports it is apparent that participants would benefit from being able to interrogate the wound in real-time with the patient present. Whilst access to the clinical history would facilitate clinical diagnoses by eliminating guesswork. One participant suggested in their report that power Doppler might enable better quantification of wounds due to its ability to detect vascular changes associated with certain wound associated factors such as Charcots’ neuroarthropathy and osteomyelitis [30, 31]. In addition, the utility of elastography to characterise soft tissue stiffness might help with the identification of characteristics such as sinus tracts which would appear softer in appearance by elastography than their surrounding tissues.

Specific reporting patterns were identified from DFU from static b mode sonographic images. All podiatrists discussed observational findings using the superficial to deep approach before suggesting their clinical judgement. This formulaic response is likely a result of training in ultrasound report writing. This approach may not be optimal when looking at the less organised tissues that results from a wound, and perhaps a more lesion-centric approach, akin to that adopted when reporting breast tumours, would enhance the quality of reporting [32].

The terms and language used by participants to identify specific morphology of DFU soft tissues was variable. The development of a robust set of definitions for DFU morphology in sonographic images would improve reporting practices. Establishing a standard lexicon for describing the sonographic characteristics of wounds is an important step in establishing consistency in clinical practice. Using the most frequently reported terms from this scoping exercise, a preliminary glossary of terms which pertain to wound ultrasound have been formulated (Glossary 1).

### Glossary 1. Glossary of terms for use in wound USI

#### Future directions

In practice there is little evidence to show the impact and effect of USI on DFU management. Although, podiatrists with USI skills have the potential to integrate this technique as part of their wound management practice, there is acknowledgement that there is a need for the development and validation of definitions, reference values and protocols in order to safely implement into clinical practice. Further understanding is required around evidencing the benefits, outcomes and impact of this technique in relation to patient care. We envisage the technique could be accessible in community and multidisciplinary care services to support treatment decision making, could reduce service line costs for clinical teams by reducing imaging demand in secondary care services and reduce the number of appointments. For patients, we hypothesise that this technique of ‘looking into the foot’ could be used as an educational tool to encourage patients to adopt self-care practices and monitor their feet. This has approach has already been adopted in the field of rheumatology [33].

#### Limitations of this scoping exercise and future directions

The authors acknowledge some limitations to this present scoping exercise. Firstly, participants were not given an extensive patient medical history that would ordinarily be available in clinical practice. However, our intentions being not to encourage podiatrists to use their clinical diagnoses skills but their USI interpretation skills to comment on the report. Still scans were used for the scoping exercise rather than dynamic scanning to capture anatomical assessment and interpretation, and this could have made it difficult for podiatrists to interpret the scans. The authors accept that more information from dynamic scanning across a wider field of view would enable more accurate assessment of tissue quality and possible artefact, in relation to the foot structures being assessed. Future research should take into consideration the additional feedback from the participants that a single static image hampered their ability to adequately report the wounds. Conducting a USI examination in real time would enable more comprehensive interrogation of the target area compared to viewing only a single retrospective scan. For reliability and validity, real time scanning with interpretation would reflect current practice and likely improve reporting accuracy.

An additional limitation is the lack of ‘gold standard’ against which to ‘benchmark’ the reports provided by the participants. There is currently no agreement on the reference points related to wound sonography. Established values need to be considered when scanning a DFU, hence the importance of validation, training and establishment of protocols. The authors appreciate this is a new application and that further study is required to determine the training aspects needed to build capacity in USI examination of foot wounds. This work provides early insight into the potential application of USI and has identified some key recommendations pertaining to validation, protocol development and training that are necessary to enable the adoption of USI into wound assessment in clinical practice (Recommendations 1).

### Recommendations 1: recommendations to assist the translation of USI wound assessment into clinical practice

## Conclusions

This scoping exercise has shown that podiatrists from two different countries, with varying ultrasound training and experience are able to translate their existing USI skills to make rudimentary reports on the clinical features of DFU. Whilst some reported findings were similar between podiatrists, especially in the domain of bone-related findings, interpretation of soft tissues seen in wounds were more variable, suggesting that the establishment of accuracy and reliability is needed as a validation of this novel use for USI. Further work, including the development of terminology, protocols and training materials will enable USI techniques to be applied in routine clinical practice for the purpose of wound assessment. This will enable safe, accurate and detailed reporting of DFU at the point-of-care as and more comprehensive and timely management of DFU, with the potential for adoption in other wound types such as venous leg ulcers and pressure injuries.

## Data Availability

The anonymised data that support the findings of this study are available from the corresponding author upon reasonable request.

## Abbreviations

3D: Three dimensional
AUS: Australia
CASE: Consortium for the Accreditation of Sonographic Education
CT: Computerised tomography
DFU: Diabetic Foot Ulcer
DM: Diabetes Mellitus
FHL: Flexor Hallucis Longus
MRI: magnetic resonance imaging
MSK: Musculoskeletal
OM: Osteomyelitis
PET: positron emission tomography
PTB: Probe-to-bone
UK: United Kingdom
US: Ultrasound
USI: Ultrasound Imaging
